# A Multi-Sensor System for Silkworm Cocoon Gender Classification via Image Processing and Support Vector Machine

**DOI:** 10.3390/s19122656

**Published:** 2019-06-12

**Authors:** Alex Noel Joseph Raj, Rahul Sundaram, Vijayalakshmi G.V. Mahesh, Zhemin Zhuang, Alessandro Simeone

**Affiliations:** 1 Department of Electronic Engineering, Shantou University, Shantou 515063, China; jalexnoel@stu.edu.cn (A.N.J.R.); zmzhuang@stu.edu.cn (Z.Z.); 2 Department of Agricultural and Food Engineering, Indian Institute of Technology Kharagpur, West Bengal 721302, India; rahulks@iitkgp.ac.in; 3 Department of Electronics and Communication Engineering, Dr T Thimmaiah Institute of Technology, Kolar Gold Fields, Karnataka 563120, India; vijayalakshmi@drttit.edu.in; 4 Intelligent Manufacturing Key Laboratory of Ministry of Education, Shantou University, Shantou 515063, China

**Keywords:** multi-sensor, image processing, support vector machine, pattern recognition

## Abstract

Sericulture is traditionally a labor-intensive rural-based industry. In modern contexts, the development of process automation faces new challenges related to quality and efficiency. During the silkworm farming life cycle, a common issue is represented by the gender classification of the cocoons. Improper cocoon separation negatively affects quantity and quality of the yield resulting in disruptive bottlenecks for the productivity. To tackle this issue, this paper proposes a multi sensor system for silkworm cocoons gender classification and separation. Utilizing a load sensor and a digital camera, the system acquires weight and digital images from individual silkworm cocoons. An image processing procedure is then applied to extract significant shape-related features from each image instance, which, combined with the weight data, are provided as inputs to train a Support Vector Machine-based pattern classifier for gender classification. Subsequently, an air blower mechanism and a conveyor system sort the cocoons into their respective bins. The developed system was trained and tested on two different types of silkworm cocoons breeds, respectively CSR2 and Pure Mysore. The system performances are finally discussed in terms of accuracy, robustness and computation time.

## 1. Introduction

**Figure 1:**
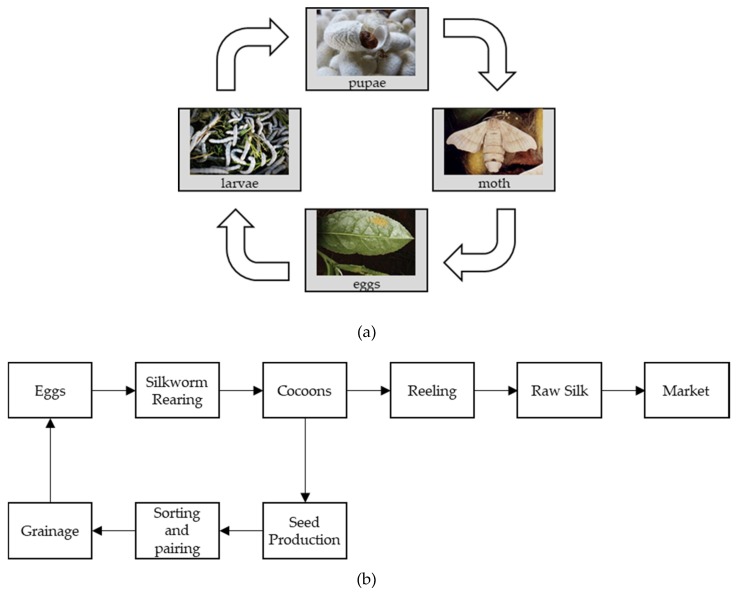
(**a**) Silkworm life cycle, (**b**) silkworm process flow for commercial usage.

Silk is the most distinguished textile in the world. It has a natural luster and is popularly known as the “Queen of Textiles” [1]. Silk is a product of sericulture, which is produced from silkworm or Bombyx mori, which means “silkworm of the black mulberry tree” [2,3]. Like most insects, the silkworm life cycle has four stages of development, respectively egg, larva, pupa, and adult moth (Figure 1a). A pair of male and female fully-grown adult moth mate with each other, and the female subsequently lays eggs and dies. The egg hatches and emerges out as a larva (also called a caterpillar), which feeds on mulberry leaves and grows for a period of 4 weeks. Once the larva stops feeding it enters the pupal stage, where it uses its secretion to form a protective outer shell called a cocoon [4]. After 3 weeks, the chrysalis emerges from the cocoon as a moth, it mates, and the female lays eggs permitting the life cycle to restart. Among these four stages, cocoons are of commercial importance since a continuous filament of raw silk is directly produced from cocoons by terminating the growth of the caterpillar while inside the cocoon.

The sericulture industry is labor intensive, mostly rural-based, and multidisciplinary in nature. It involves on-farm activities such as mulberry cultivation, egg production, silkworm rearing, cocoon production, and off-farm activities like raw silk reeling, spinning, throwing, and weaving. Grainage centers [5] separate the cocoons based on their gender and allow pairs to mate and produce silkworm eggs (also called seeds). These seeds are utilized by the farmers for cocoon production and later based on the quality and requirements, the cocoons are either sent for reeling, to obtain raw silk or made available to the grainage centers for increased seed production. 

In terms of productivity, the success of the silk industry depends mainly on the pre-cocoon stages of the silkworm life-cycle [6], however, literature and industrial practices surveys highlight a gap in the automation development in the pre-cocoon stage compared to post-cocoon stage.

Table 1 summarizes the commercially-available machinery for both pre- and post-cocoon stages, the latter of which can rely on automated machines to reduce the manual labor involved and to improve the production yield.

In terms of quality and quantity, best practices in seed production require (a) sorting the silkworms based on gender and (b) and allowing the best pairs to mate in a constrained environment [5]. Although silkworms can be sorted at different stages during their life cycle (see Table 2), the cocoon stage is the most effective since it causes minor damages to the pupa [6].

**Figure 2:**
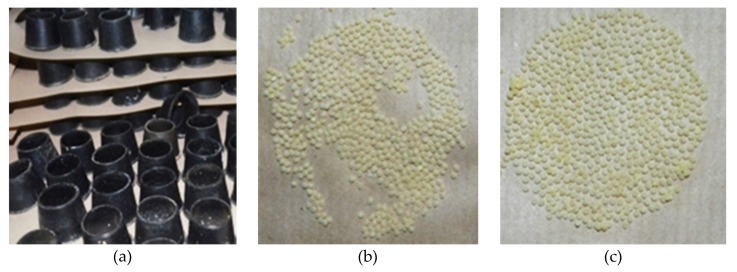
(**a**) Image of cubicles used for egg production. (**b**) Eggs produced by incorrect separation of cocoons. (**c**) Eggs produced by pairing best male and female cocoons.

Currently, at the grainage centers [7], the cocoons which contain the live chrysalis are weighed and separated as males or females. Then the best cocoon pairs are kept in circular cubicles (Figure 2a) to subsequently allow the moth to emerge, mate and lay the eggs. This manual cocoon-sorting is made possible since females are bulkier and heavier than the male chrysalises. However, this task is still mainly performed manually by employing highly skilled professionals, since a low accuracy in cocoon separation according to the gender may lead to selfing [8,9], decreasing the quantity of the laid eggs [10].

Moreover, the quantity of eggs depends on the proper selection of cocoons quality, in terms of weight and shape, which should fall in specific ranges indicated by industrial standards [6,10]. According to the industrial partner best practices, the weight of Pure Mysore-breed silkworm cocoons ranges from 0.8 to 1.1 g for male and from 1.2 to 1.4 g for female cocoons. The CSR 2 breed cocoons weight ranges from 0.7 to 1.4 g for male and 1.5 to 2.0 g for female cocoons. Moreover, overall circumference of the female cocoon is larger when compared to the male cocoons.

Figure 2b shows the effect of selfing due to incorrect classification on the quantity of laid eggs compared to the ideal separation (Figure 2c).

**Figure 3:**
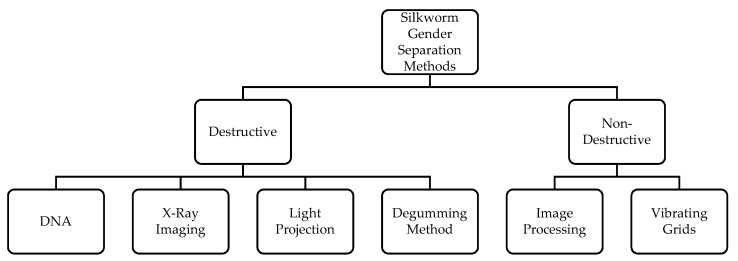
Methods of silkworm gender separation process.

The literature review provides detailed information about various methods and techniques used for gender separation of silkworm cocoons with a focus on feasibility of usage at grainage centers by untrained professionals. The available techniques can be broadly classified as destructive and non-destructive methods. Destructive methods effectively differentiate the silkworms, but cause permanent damage either to egg, larva, cocoon, or pupae and therefore cannot be further used for seed production or reeling. Non-destructive methods cause less or minimal damage to the silkworms and allow the insect (egg, larva, cocoon, or pupae) to be used in subsequent process. Figure 3 illustrates these methods and the following paragraph reports them in detail.

A DNA based gender separation method for sorting silkworm at larvae stage was developed by Tang Pei [15]. It is a chemical based method which required highly trained professionals and suitable only for constrained working environments. X-ray imaging-based gender separation techniques were presented in [14,16]. Calvin J. Witdouck [14] passed X-rays through the silkworm larvae (caterpillar) to identify its gender. Cai et al. [16] demonstrated a prototype with an appropriate classification tool for successfully discriminating the gender of silkworm by X-ray imaging the cocoons. Shape features such as major axis, minor axis, ratio of major axis to minor axis, eccentricity, roundness, rectangularity, complexity, concave and convex characteristics of the chrysalis are extracted from X-ray images and inputted to pre-trained classifiers such as k-Nearest Neighbor (kNN) [17], Linear Discriminant Analysis (LDA) [18], Neural Networks (NN) [19] and Support Vector Machine (SVM) [20] to accurately classify the cocoons as male or female. The authors considered 1071 samples from three hybrid breeds and have reported an accuracy of 93.68% with kNN classifier. Although the X-ray-based methods provide considerably high classification accuracy, constant exposure of the silkworm cocoons to the X-rays may again result mutations leading to poor egg lays, if the cocoons are used in grainages for seed production [21].

A number of light-based sorting systems were developed by Shinji Hayashizaki et al. [22], Raie et al. [23], Yang Bin et al. [24]. In [22] the authors reported light inspection system where the cocoon was cut-open and each pupa was examined under visible light and near infrared light of wavelength 600 nm to 1100 nm. Further the frequency data reflected from the pupa are analyzed and compared with the predefined threshold, based on which the pupa was classified as male or female. Further, in [23], each pupa was again examined under a stereo-microscope. These images were transferred to a computer for processing and image analysis. Under analysis, the gender gland of the silkworm was observed, based on which the sorting was carried out. In both cases, the cocoons are opened to take out the pupa resulting in a high chance of the pupa getting damaged, and further cut cocoons cannot be used for reeling. Moreover, the method is slow and requires trained professionals to examine each pupa accurately. 

Zhang et al. [25] developed an apparatus to classify male and female cocoons by radiating ultraviolet rays on the cocoons. The cocoon being tested presented different fluorescence characteristics based on gender. The male cocoon showed yellow (wavelength in the range of 577–597 nm) and female cocoon showed purple (wavelength 390–455nm) fluorescence characteristics respectively. The entire process has to be carried out in a dark room and the assessment is based on the human vision. Additionally, this method is labor intensive and requires additional overhead of 200 W power source and 3600 A wavelength UV light source. This method was further improved by Yang Bin et al. [24] who automated the process based on the color assessment of the fluorescence characteristics provided by male cocoons, integrating a photosensitive yellow filter with the ultraviolet filter for detecting and separating the male cocoon from the female ones.

Yu Xiaohua [26] developed a method for identifying male silkworm cocoon by degumming, i.e., soaking the cocoon in hot water and extracting the fibers. Later the fibers are subject to a chemical process to assess the methionine content and aspartic acid value of three amino acids, based on which the gender can be determined. This is again a non-automated chemical-based technique, which requires heating of the cocoon which can damage the pupa.

Very few non-destructive methods were developed for gender separation of silkworms. An MRI imaging technique operating at 20MHz was used by C. Liu, Z.H. Ren [27] to determine the gender of the silkworm. The MRI image of the cocoon along with live pupa is acquired and later transformed by fast Fourier transform and T2 weighted images (to accurately reflect the tissue contrast into picture contrast) were obtained that aids in distinguishing the gender of the silkworms. Although the method is non-destructive and causes minimal damage to cocoon and the pupa, the imaging process is expensive and practically unsuitable for grainage centers.

Udaya et al. [28] developed a prototype which included metal grids of various sizes which vibrate when connected to an electric motor. The cocoons were sorted based on the size of the cocoons. The female cocoons are bulkier whereas males are thin and slender. The cocoons are transferred into the vibrating grids of the sorting machine which are of varying size to separate the cocoons. Though the system was able to achieve an accuracy of 96% in sorting, the device is not meant for gender separation but mainly used for grading the quality of Tasar variety of cocoons. The graded cocoons are later sent to reeling where raw silk is extracted from the graded cocoons. 

Further, Mahesh et al. [20] proposed a novel non-destructive vision-based system to classify the cocoons. The methodology integrated the weight, volume, and ZM-based shape features of the cocoons to form an integrated feature vector for training kNN, LDA, NN, and SVM classifiers. To validate the integration of these features, the performance was compared with the one obtained from integration of geometric shape features and integration of weight and volume with geometric shape features. The method used CSR2 and pure Mysore breeds of cocoons to conduct the experiment. The results indicated a better performance of NN and SVM classifiers. An accuracy of 91.3% was achieved from CSR2 cocoon with NN classifier and 100% from pure Mysore cocoons via SVM-based classifier.

This detailed literature review indicates the existing technologies used for silkworm gender separation at different stages of their life cycle. Separation of silkworm at stages such as eggs, larvae, or pupae is not feasible in grainage centers [27,29]. X-ray or MRI images of the cocoon are high-cost alternatives which provide accurate classification, but the radiation can damage the pupa inside the cocoon. Currently, at grainage centers, the sorting process is manual, where the cocoons’ weight (which includes the live pupa) and shape are used as features to distinguish their gender. 

Taking into account literature and industrial practice gaps, this paper presents the design and development of a novel non-destructive multi-sensor-based system to classify silkworm cocoons according to their gender. The system extracts the features of cocoons (weight and shape) individually and provides them as inputs to a pre-trained pattern classification model which in turn classifies the cocoons as male or female. Subsequently, a pair of air blowers and a conveyor system sort the cocoons into their respective bins. The developed system was trained and tested on two types of silkworm cocoons breeds, namely CSR2 and Pure Mysore, both provided by Central Silk Board Registered Grainage Center, Karnataka, India. 

The prominent advantages of the developed system are (a) elimination of human intervention in separation process, (b) reduction in mis-classification error, (c) good repeatability when compared to manual separation process, and (d) overall increase in speed of separation process.

## 2. Design and Development of Silkworm Cocoon Gender Classification Multi-Sensor System

**Figure 4:**
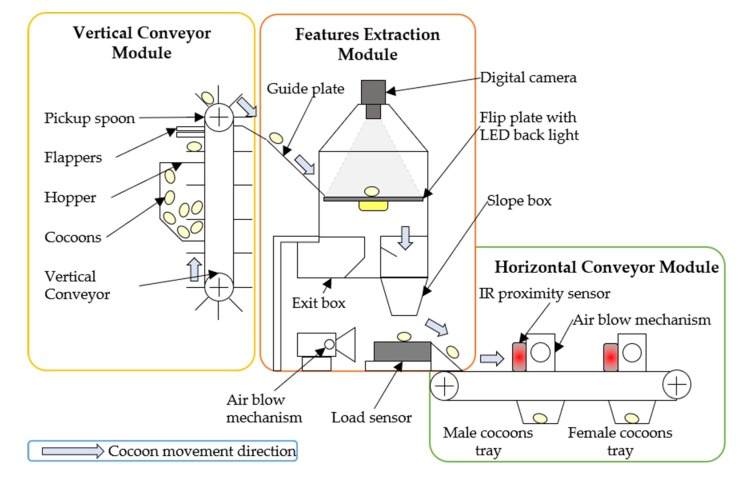
Schematic of silkworm cocoons separating machine.

**Figure 5:**
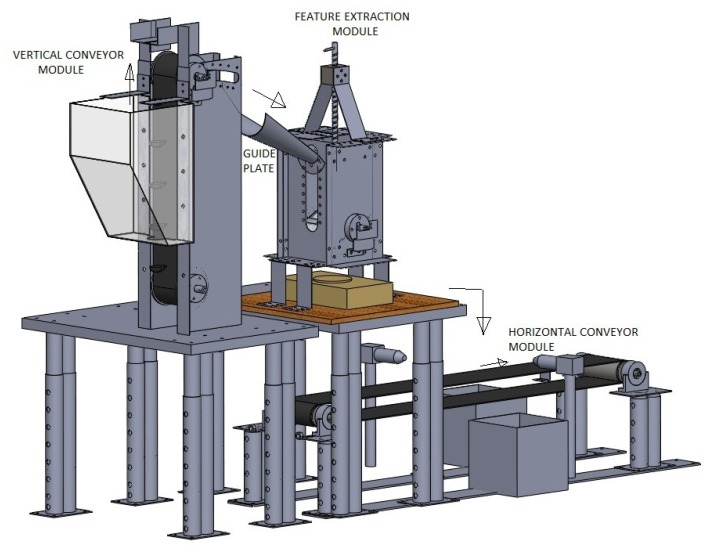
3D model of silkworm cocoons separating machine.

**Figure 6:**
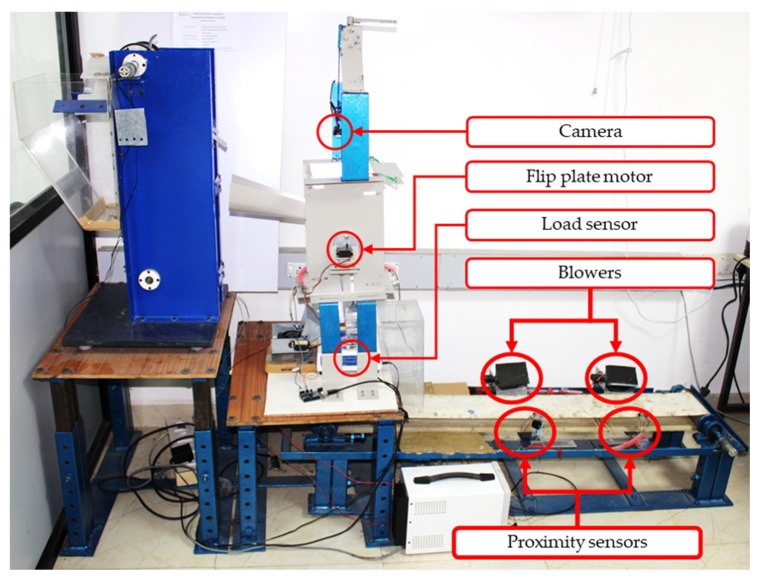
Developed prototype of silkworm cocoons multi-sensor classification system.

The multi-sensor system was designed and prototyped with the aim of performing automatic silkworm cocoons gender classification process. A schematic diagram of the proposed system is shown in Figure 4. The corresponding 3D model and developed prototype are shown in Figure 5 and Figure 6. 

The cocoons were initially stored in the hopper, then individually picked by a vertical conveyor module (VCM). The cocoons entered one by one into the feature extraction module (FEM), where each cocoon was analyzed and their features such as shape and weight were extracted. A dedicated software, which executes on a standalone workstation, acquired a digital image and weight of each cocoon, and subsequently extracted significant shape-related features from image instances. Image features and weight data were then combined in an input feature vector, which was inputted to a pre-trained pattern recognition classifier for decision-making on gender classification. Eventually, individual cocoons were transported through a horizontal conveyor module (HCM) which performed the physical sorting of the cocoons and disposed them into dedicated male or female bins. Each module is illustrated in detail in the remainder of this paper.

### 2.1. Vertical Conveyor Module (VCM)

**Figure 7:**
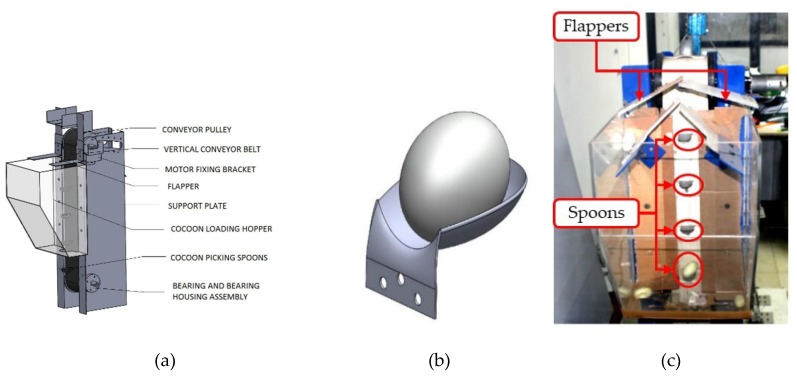
(**a**) 3D model of vertical conveyor module (VCM) module; (**b**) 3D model of spoon carrying a cocoon; (**c**) prototype of VCM module.

**Figure 8:**
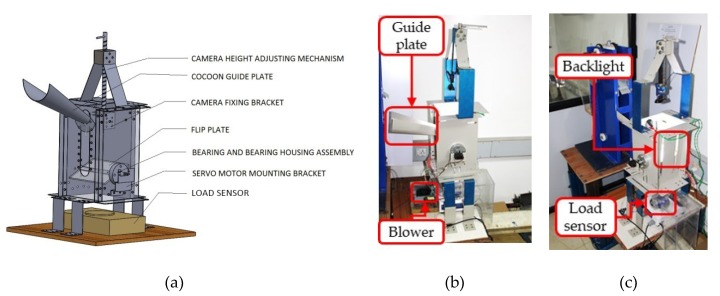
(**a**) 3D model of FEM; (**b**,**c**) prototype of FEM shown in two different angles.

The purpose of VCM is to pick individual cocoons from the hopper and feed them into the feature extraction module at a constant velocity without causing major physical damage to cocoons. It consists of a 60 cm-long conveyor belt, which passes through a 12 cm-diameter pulley mounted on the frame plates with the help of bearing support (Figure 7a,c). A 12 V, 10 rpm DC motor drives the pulley which allows each cocoon to travel at a speed of 6.3 cm/s. A loading hopper made from acrylic, which can accommodate up to 1 kg of cocoons (approximately 770 specimens), is rigidly mounted on frame plates. The VCM is endowed with 16 specially-designed concave-shaped spoons that can accommodate one cocoon at a time. Such spoons are riveted on the conveyor belt as shown in Figure 7a,c. The distance between two consecutive spoons is 10 cm. Spoon edges are smoothed to avoid sticking to the cocoon fibers. Two pairs of flappers are mounted on both sides of the metal frame (Figure 7c). The position of flappers was determined experimentally to align the cocoons with the concavity of the spoons and to avoid clinging to the fibrous outer shells of the other cocoons. Once the cocoons were picked up by these spoons, they were transferred to FEM for analysis. In this respect, a guide plate (Figure 8a,b) is positioned on rear side of VCM to enable smooth transfer of cocoons from VCM to FEM. 

### 2.2. Features Extraction Module (FEM)

The FEM shown in Figure 8 consists of a camera support bracket, flip plate, slope box, exit box, a detachable load sensor, and an air blower mechanism. The module extracts both the shape features of cocoon via image processing and the weight of the cocoon (in g) via the load sensor and feeds such information into a binary classifier to determine the cocoon gender. A 5 mega pixel digital camera is mounted on the support bracket and a flip plate made of acrylic is mounted below the camera assembly. The structure allows continuous acquisition of objects on the flip plate. The flip plate is attached to a rotating shaft which is powered by a servo motor to allow the plate to be positioned at three different orientations (a) 0° (horizontal), (b) 90° (clockwise), and (c) −90° (counter clockwise). When the system is switched on, the flip plate is positioned horizontally to receive cocoons from the VCM. 

**Figure 9:**
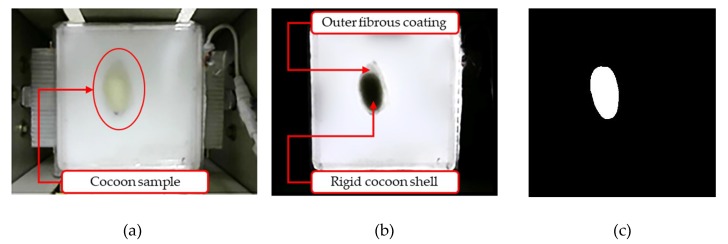
(**a**) RGB image of the cocoon on flip plate captured by FEM camera without backlight, (**b**) RGB image of the cocoon on flip plate captured by FEM camera with backlight illumination, and (**c**) binarized image of the cocoon shell with fibrous outer surface removed.

Silkworm cocoons have a hard shell that is covered by fibrous outer coating as shown in Figure 9a,b. The accuracy of the classifier depends on how well the shape features are extracted from the cocoon rigid shell images by eliminating the negative effect of fibrous coating. Practically, it is not possible to remove the fibers from each cocoon manually at grainage centers before loading cocoons into the hopper. To tackle this issue, the FEM is endowed with an 18 W square LED panel light (shown in Figure 9a,b) attached to the flip plate. Such an illumination system provides the necessary back-lighting to capture the silhouette of the hard shell of the cocoon enabling accurate area calculation by image thresholding techniques [30,31]. 

By comparing the two images, the cocoon sample in normal light conditions without backlight (Figure 9a) and the same cocoon sample placed on the flip plate with backlight (Figure 9b), the advantage of the adopted illumination system is evident in the results.

After image acquisition, the cocoon is transferred to the load sensor by letting it fall through a slope box (see Figure 4), where the velocity of falling cocoon is attenuated by travelling through number of inclined slopes. Once fallen on the load sensor unit, the cocoon weight data are acquired with a resolution of ±0.01 g and transferred to the workstation.

**Figure 10:**
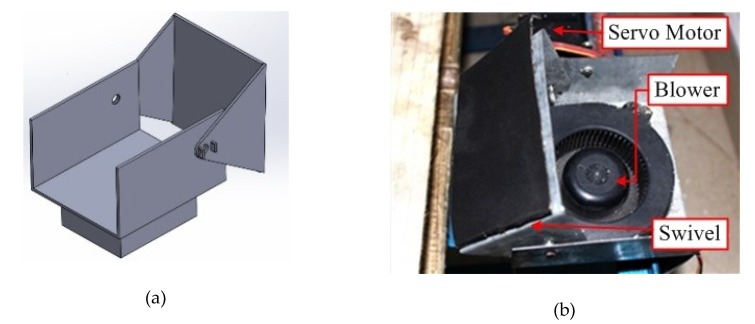
(**a**) 3D model of air blower frames; (**b**) developed air blower mechanism.

Following the weight data acquisition, an air blower mechanism (Figure 10) is employed to transfer the cocoon from the load sensor unit to the HCM. The blower mechanism consists of an air blower which continuously provides a compressed air supply and a freely rotating swivel arm which is used to stop the air flow instantly. One end of the swivel arm is coupled with a servo motor and the other end is fixed on the side wall by a freely rotating cylindrical pin joint. Figure 10a shows the mechanism in closed position (the air stream is blocked). Figure 10b shows the mechanism in open position (the air stream is directed to the cocoon). Normally, the air blower mechanism is in the closed position, hence no air flow is directed on the cocoon. Once the weight data are acquired, the system sends a command to the servo motor and the swivel arm opens for 2 s allowing for the cocoon to be transferred from the load sensor to the HCM. As regards the FEM power requirement, the major contributions are represented by the back light: 18 W, the flip plate servo motor: 5 V × 0.9 A = 4.5 W and the digital camera: 5 V × 0.5 A = 2.5 W, for a total power requirement equal to 25 W.

### 2.3. Horizontal Conveyor Module

**Figure 11:**
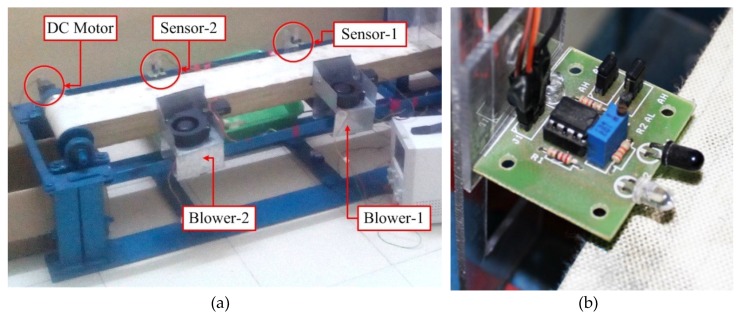
(**a**) Horizontal Conveyor Module; (**b**) IR proximity sensor close-up.

Once the shape and weight features are extracted by the FEM, the cocoon moves to next module, The Horizontal Conveyor Module (HCM) consists of a rotating conveyor belt, an infra-red (IR) proximity sensors and 2 blower mechanism units (Figure 11). The conveyor belt is 2-meter-long and actuated by a 12 V DC motor (10 rpm and 120 kg·cm torque) to rotate continuously at a speed of approximately 8 cm/s. A pair of IR sensors and a pair of air blowers are placed at a distance of 40 cm along the conveyor as shown in Figure 11. 

Their positions were determined empirically based on the computation time required by the workstation to provide the classification index. The HCM classifies the cocoons based on the index obtained from the workstation. As the cocoon crosses the first sensor–blower pair, its index is retrieved from the workstation. Based on the predicted index, the blowers in each pair activate/deactivate and transfer the cocoon to their respective trays.

### 2.4. Communication and Synchronization of Modules

**Figure 12:**
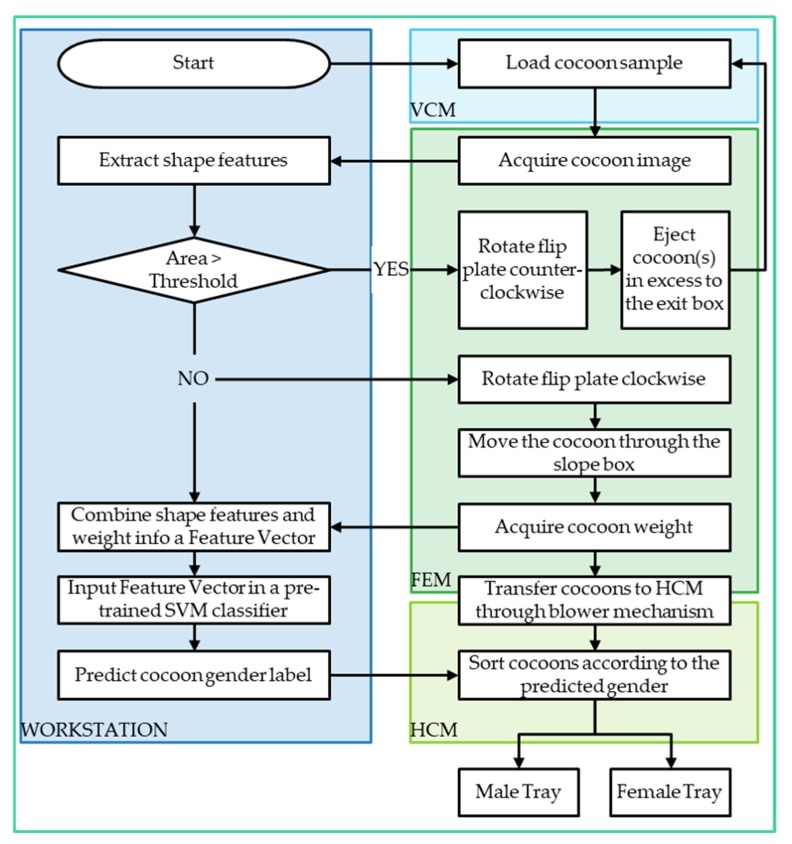
Flow diagram of silkworm cocoons gender classification machine.

The prototype consists of three modules which contain several individual components. Each component performs an individual function to carry out the cocoon sorting process. These components need to be synchronized in order perform automated operation. Figure 12 presents the flow diagram of silkworm cocoons gender sorting machine. 

The acquired cocoon image in FEM module is sent to the workstation, where the shape features (area, perimeter, major axis length, minor axis length, etc.) are computed. At times, there is a chance of entering more than one cocoon into the FEM module. This condition is detected by computing the cocoon area from the binarized image and comparing it to an empirical threshold value. The experimental setup was designed to achieve the most favorable experimental conditions in terms of image quality and illumination, for this reason, the thresholding operation for image binarization is carried out on the acquired image utilizing the Otsu’s algorithm [32]. If the computed area exceeds the threshold, then exceeding cocoons are ejected by rotating the flip plate in counterclockwise direction. Ejected cocoons move out of the module through exit box (Figure 4) to be fed back to the hopper. If the binarized image area results within the threshold limit (i.e., only one cocoon present on the flip plate), a signal is provided to the microcontroller to rotate the flip plate in clockwise direction to transfer the cocoon to the load sensor smoothly through slope box. At this point, the sample cocoon weight is acquired and provided to the algorithm in the workstation. Shape and weight features are then combined and fed to a pre-trained SVM to determine the gender of cocoon under examination. Further, the cocoon index and its corresponding predicted label are stored in the workstation and the cocoon present on the load sensor is moved to HCM module by the air blower mechanism.

As the cocoon moves along the HCM, the IR proximity sensors (Figure 11) provide an input signal to microcontroller-2 which in turn retrieves the classification label of the current cocoon obtained from the workstation. The label is used to control the respective blowers. In this respect, if the predicted label is “male”, the first blower is triggered, and the cocoon is pushed on to the “male cocoon tray”. Conversely, if the cocoon label is “female”, the second blower is triggered and pushes the cocoon into the “female cocoon tray”.

## 3. Experimental Methodology

The experimental campaign was carried out on two silkworm cocoons breeds, namely CSR2 and Pure Mysore, both provided by the industrial partner. Prior to the experimental tests’ commencement, the cocoons were manually labelled as male and female by highly trained and skilled professionals using a weight threshold as discriminating parameter. The cocoon weight is an important factor since it is highly correlated to the cocoons gender (i.e., cocoons above the weight threshold are considered as females and ones below are males) and is most commonly used gender separation method employed in the grainage centers. A weight threshold of 1.4 g for CSR2 breed and 1.1 g for Mysore breed was used to separate the cocoons as male and female to build the ground truth used for benchmarking. Besides cocoon weight, shape features are also significant in discriminating the cocoons based on gender [20]. 

A total number of 167 cocoons was used to build the dataset, which included 76 Pure Mysore and 91 CSR2 breeds. For Pure Mysore breed, there were 35 male and 41 female specimens; similarly, CSR2 breed contained 47 males and 44 females. The training set was used to pre-train the SVM classifier for decision-making on gender classification. The dataset subdivision was carried out using the hold-out method [33] with the following proportions: 60% for training and 40% for testing, as shown in Table 3.

The training set was used to pre-train the Support Vector Machine (SVM) classifier. SVM is based on statistical learning theory aimed at determining the location of decision boundaries yielding the optimal separation of classes [34]. For a binary pattern recognition problem in which the classes are linearly separable the SVM selects from among the infinite number of linear decision boundaries the one that minimizes the generalization error. Thus, the selected decision boundary will be one that leaves the greatest margin between the two classes, i.e., the sum of the distances to the hyperplane from the closest points of the two classes [35]. The data points that are closest to the hyperplane are used to measure the margin; hence these data points are termed “support vectors” [29].

If the two classes are not linearly separable, the SVM tries to find the hyperplane that maximizes the margin while, at the same time, minimizing the misclassification errors. SVM can also be extended to handle nonlinear decision surfaces by projecting the input data onto a high-dimensional feature space using kernel functions [36] and formulating a linear classification problem in that feature space. In this research work a linear kernel has been utilized to train the SVM.

In order to train the classifier, the separated training cocoons were labelled and indexed manually prior to being loaded into the VCM hopper. Once the cocoon was transferred from the VCM to FEM, the cocoon’s silhouette was acquired by camera and passed to the workstation. If the camera was rigidly fixed at distance of 18 cm from the flip plate, the area of an individual cocoons ranged between 500 and 550 pixels. If the area was greater than this interval, the system assumed that the VCM has transferred more than one cocoon to FEM, therefore the exceeding cocoons were ejected back to the hopper through the exit box. Once the FEM ensured the feeding of a single cocoon, a number of shape features were computed and extracted from the silhouette image as reported in Table 4.

Once the features were computed, the flip plate rotated clockwise and weight of the cocoon (W) was obtained from the weight sensor placed below and serially transferred to work station. Later, using the air-blower mechanism explained in section, the cocoon moved to the next stage of the pipeline. At the work station extracted shape features were integrated with the weight to form an integrated feature vector (*IFV*) as shown in Equation (1):

(1) $$IFV\;=\;\left\{W,\;A,\;P,\left(\frac{\lambda_{1}}{\lambda_{2}}\right),\;E,\;C,\;R,\;S,\;A_{C}\right\}$$

The IFV was further normalized to standardize the range of obtained features using Z-score normalization [37], given by:

(2) $$IFV_{norm}=\frac{IFV_{i}-\mu_{i}}{\sigma_{i}},\;i=1,\ldots ,L$$

where L is the length of the *IFV*, μ is the mean of the feature and σ the standard deviation of the feature. The normalized *IFV* is labeled for supervised machine learning. Label “1” indicates category “Male” and “0” indicated category “Female”. 

## 4. Results and Discussion

The proposed pattern classifier performance is assessed in terms of accuracy, robustness and computation speed.

### 4.1. Classifier Accuracy

**Figure 13:**
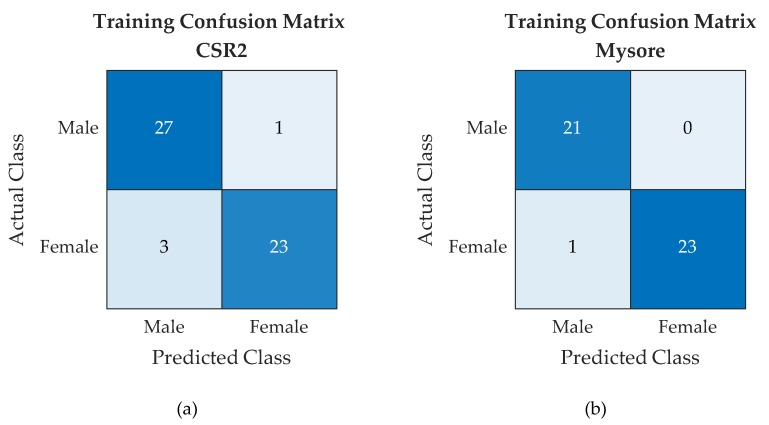
SVM training confusion matrices for CSR2 (**a**) and Pure Mysore cocoons (**b**).

The performance assessment of the SVM classifier is carried out using the hold-out method [33], where the labelled training set is used in training the classifier to create an optimal model. The model is further evaluated using the testing data set. The results of the training process are displayed using a confusion matrix (CM) to calculate the performance metrics of the training phase [38]. Such matrices show the True Male, True Female, False Male, and False Female. With reference to the SVM training process, the classification results CMs for the CSR2 and Pure Mysore breeds cocoons are reported in Figure 13.

**Figure 14:**
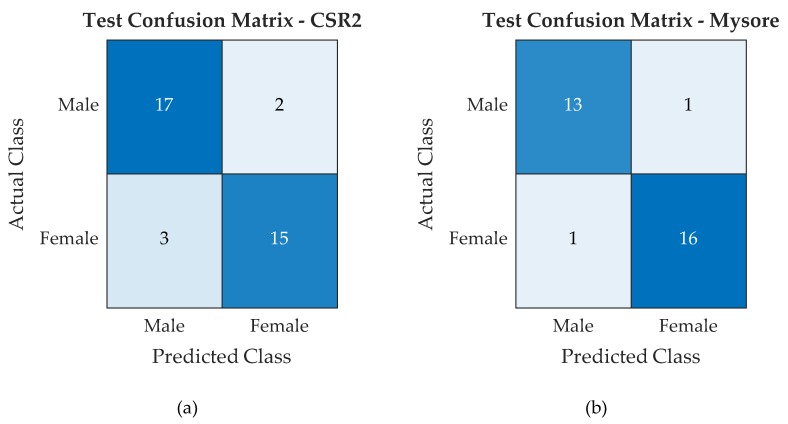
SVM test confusion matrix for CSR2 cocoons (**a**) and Pure Mysore cocoons (**b**).

To validate the accuracy of the prototype, unknown cocoon samples are indexed and loaded into the VCM’s hopper. As the cocoon travel from VCM to FEM, its features are extracted and transferred to the workstation where the pre-trained SVM provides the predicted classification label. The label and the corresponding cocoon indices are stored as look up table within the workstation. When the cocoon moves along the HCM, the IR proximity sensors and with the microcontroller query the workstation to provide the classification label of the current cocoon. This label is used by the sensor to blow the cocoons to their respective trays as explained in Section 2. This process utilizes all the cocoons present in the testing dataset and the performance of the prototype with SVM model is evaluated similar to that of the training process. With reference to the SVM test process, the classification results CMs for the CSR2 and Pure Mysore breeds cocoons are reported in Figure 14.

From the confusion matrices reported in Figure 13 and Figure 14, a number of performance metrics were computed, namely Accuracy, True Male Rate (TMR), True Female Rate (TFR), Male Predictive Rate (MPR), Female Predictive Rate (FPR), and F1 score [39,40]. Such performance metrics are reported in Table 5 with reference to both training and test phases for CSR2 and Pure Mysore cocoons respectively:

### 4.2. Robustness and Computation Speed

**Figure 15:**
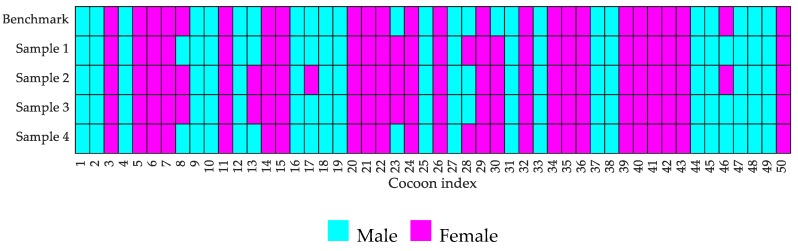
Graph of cocoon index vs. predicted gender for the sample batch.

To validate the prediction robustness, 50 cocoons were randomly selected from CSR2 breed and four trials were conducted. The cocoons were indexed, and their classification labels recorded prior to the trial conduction. The prediction results from protype each cocoon for all the four trials are illustrated in Figure 15. The chart shows that 44 cocoons were correctly predicted in all the four trials. Hence the repeatability of the machine is calculated as the ratio of the number of correctly classified cocoons in all the four trials over the total number of tested cocoons. The repeatability of cocoons separation through the fusion of weight and shape features using the prototype resulted to be 44/50 = 88%.

The average time required for a cocoon to reach the FEM from VCM is 4.6 s. The cocoon then lays on the flip plate for about 1 s to allow for the image acquisition. The cocoon is subsequently transferred to the load sensor, where the weight feature extraction requires 1.2 s. In total, the cocoon stays in the FEM module for about 2.2 s. The cocoon reaches the collection tray through the HCM in about 3.6~4.1 s. Thus, the maximum time taken for a cocoon to reach the tray from hopper is 10.9 s.

From the observed results, the proposed system classifies approximately 5.5 cocoons in a minute and thus it can classify 330 cocoons in an hour. For eight hours shift, the prototype can classify about 2640 cocoons. Considering an average weight of a cocoon equal to 1.3 g, the presented system can classify approximately 3.4 kg of cocoons in eight hours shift yielding an accuracy ranging from 86.48% to 93.54% depending on the breed, whereas highly experienced staff working in a grainage center can probably sort a similar daily amount of cocoons bearing in mind that manual classification is prone to human error over prolonged working hours and may lead to serious health and safety issues [41,42]. 

## 5. Conclusions

The work presented in the paper represents a kick-off in modern sericulture automation to eliminate human intervention in classifying silkworms based on gender in the cocoon stage without damaging the shell. The developed system has the potential to boost the productivity of grainage centers who are currently carrying out the gender classification process manually. The results obtained during testing showed a maximum accuracy of 93.54% with a repeatability of 88%, demonstrating a potential suitability of the proposed method for industrial applications.

Future research efforts need to be focused on the following critical aspects for improving the industrial suitability of the system:

- design optimization to reduce the overall dimensionality and operation speed from a hardware perspective in terms of more powerful workstation and more efficient blower mechanism;
- endowing the VCM with a deflossing unit [5] in order to remove the fibers of the cocoon to avoid clinging phenomena which drastically reduce the system speed;
- extend the experimental campaign to a wider variety of cocoon breeds to improve the system generalization and to increase the system versatility.

## Figures and Tables

**Table 1:** Available machinery for pre and post-cocoon stage.

Pre-Cocoon Stage	Post-Cocoon Stage
Machine for crushing shoots	Cocoon de-flossing machine
Mulberry pruning machine	Denier detecting device in silk reeling
Litter separation machine	Long skein silk book making machine
	Pedal-operated reeling twisting machine for muga and tasar silk Reeling
	Reeling and twisting machine
	Solar-operated spinning machine
	Motorized pedal-operated spinning machine
	Wet reeling machine

**Table 2:** Methods for silkworm gender classification for different stages [11,12,13,14,15].

Stages	Methods	Remarks
Chromosome	Presence or absence of the “W” chromosome Female—ZW chromosome Male—ZZ chromosome	Not practical—high cost
Egg	Color of the egg Males are usually light yellow Females are dark brown in color	Not practical—need for skilled workers
Larval	Markings are exhibited on the larval body Female—crescent marking Males—plain	Sex separation is possible only on the 1st day of 5th instar. Process is laborious and too slow operation, larvae may get injured
Cocoon	Color and weight Color—females are golden yellow/ Males white—CSR2 Weight—females are heavier than males	Color depends on various silkworm breeds Weight—each cocoon is weighed individually and sorted—presently followed in grainages—non-destructive
Pupa	Males are smaller in size whereas females are plumper	Reliable/low error—cocoons have to be cut open to remove the pupae, which may cause injury to pupae
Moth	Males are small, slender active moving in semi-circles with bent abdomen/females are bigger with bloated abdomen and rather lethargic	Males and females are easily separated. Selfing takes place affecting the quality of the eggs, health hazards from moth dust

**Table 3:** Dataset for silkworm cocoons.

	Training Set		Testing Set	
	**M**	**F**	**M**	**F**
**CSR2**	28	26	19	18
**Pure Mysore**	21	24	14	17

M: Number of male specimens; F: number of female specimens.

**Table 4:** Shape-related features extracted from the silhouette binary image.

Parameter	Description
Area (A)	Describes the number of pixels in the region of the shape
Perimeter (P)	Provides the number of pixels in the boundary of the shape
Major axis length ($\lambda_{1}$)	Specifies the length (in pixels) of the major axis of the ellipse that has the same normalized second central moments as the region
Minor axis length ($\lambda_{2}$)	Specifies the length (in pixels) of the minor axis of the ellipse that has the same normalized second central moments as the region
Eccentricity (E)	Measure of the aspect ratio. Computed using minimum bounding box (smallest rectangle containing every point in the shape) method. $$E=\;\frac{L_{b}}{W_{b}}$$ where $L_{b}$ = Length of the bounding box and $W_{b}$ = Width of the bounding box
Circularity/Roundness (C)	Circularity ratio represents how a shape is similar to a circle. It is given by the ratio of the area of a shape to the shape’s perimeter square. $C=\;\frac{A}{P^{2}}$
Rectangularity (R)	Represents how rectangular a shape is, i.e. how much it fills its minimum bounding rectangle. It is given by: $$R=\;\frac{A}{A_{r}}$$ where $A_{r}$ = Area of the minimum bounding rectangle
Solidity (S)	Describes the extent to which the shape is convex or concave. It is given by: $S=\;\frac{A}{H}$ where H = is the convex hull area of the shape. The solidity of a convex shape is always 1
Convex area $\left(A_{C}\right)$	Specifies the number of pixels in convex image. It is given by: $$A_{C}=\;\frac{\mathrm{Perimeter}\;\mathrm{of}\;\mathrm{the}\;\mathrm{convex}\;\mathrm{hull}}{P}$$ where convex hull of a region is the smallest convex region including it.

**Table 5:** Performance metrics (PM) obtained for CSR2 and pure Mysore cocoons from SVM training and testing.

	Training		Testing	
PM	CSR2	Pure Mysore	CSR2	Pure Mysore
**Accuracy:**	0.9259	0.9778	0.8649	0.9355
**True Male Rate**	0.9642	1.0000	0.8947	0.9286
**True Female Rate**	0.8846	0.9583	0.8333	0.9412
**Male Predictive Value**	0.9000	0.9545	0.85	0.9286
**Female Predictive Value**	0.9583	1.0000	0.8824	0.9412
